# Preliminary Evaluation of Translated and Culturally Adapted Internet-Delivered Cognitive Therapy for Social Anxiety Disorder: Multicenter, Single-Arm Trial in Japan

**DOI:** 10.2196/45136

**Published:** 2023-05-05

**Authors:** Naoki Yoshinaga, Graham R Thew, Yuta Hayashi, Jun Matsuoka, Hiroki Tanoue, Rieko Takanashi, Mutsumi Araki, Yoshihiro Kanai, Alisha Smith, Sophie H L Grant, David M Clark

**Affiliations:** 1 School of Nursing Faculty of Medicine University of Miyazaki Miyazaki Japan; 2 Department of Experimental Psychology University of Oxford Oxford United Kingdom; 3 Oxford Health NHS Foundation Trust Oxford United Kingdom; 4 Department of Nursing Graduate School of Health Sciences Kobe University Kobe Japan; 5 Higashi-Omiya Mental Health Clinic Saitama Japan; 6 Ogu Mental Health Clinic Tokyo Japan; 7 Department of Psychology Teikyo University Tokyo Japan; 8 Research Center for Child Mental Development Chiba University Chiba Japan; 9 Gokiso Counseling Office Nagoya Japan; 10 Department of Psychology and Behavioral Sciences Faculty of Human Sciences Tohoku Gakuin University Sendai Japan; 11 School of Psychology Cardiff University Cardiff United Kingdom

**Keywords:** benchmarking, anxiety, social anxiety, social phobia, cognitive behavioral therapy, cognitive therapy, cross-cultural comparison, Japan, mental disorders, internet-based intervention, mobile phone

## Abstract

**Background:**

Internet-delivered cognitive therapy for social anxiety disorder (iCT-SAD), which is a therapist-guided modular web-based treatment, has shown strong efficacy and acceptability in English-language randomized controlled trials in the United Kingdom and Hong Kong. However, it is not yet known whether iCT-SAD can retain its efficacy following linguistic translation and cultural adaptation of treatment contents and implementation in other countries such as Japan.

**Objective:**

This study aimed to examine the preliminary efficacy and acceptability of the translated and culturally adapted iCT-SAD in Japanese clinical settings.

**Methods:**

This multicenter, single-arm trial recruited 15 participants with social anxiety disorder. At the time of recruitment, participants were receiving usual psychiatric care but had not shown improvement in their social anxiety and required additional treatment. iCT-SAD was provided in combination with usual psychiatric care for 14 weeks (treatment phase) and for a subsequent 3-month follow-up phase that included up to 3 booster sessions. The primary outcome measure was the self-report version of the Liebowitz Social Anxiety Scale. The secondary outcome measures examined social anxiety–related psychological processes, *taijin kyofusho* (the fear of offending others), depression, generalized anxiety, and general functioning. The assessment points for the outcome measures were baseline (week 0), midtreatment (week 8), posttreatment (week 15; primary assessment point), and follow-up (week 26). Acceptability was measured using the dropout rate from the treatment, the level of engagement with the program (the rate of module completion), and participants’ feedback about their experience with the iCT-SAD.

**Results:**

Evaluation of the outcome measures data showed that iCT-SAD led to significant improvements in social anxiety symptoms during the treatment phase (*P*<.001; Cohen *d*=3.66), and these improvements were maintained during the follow-up phase. Similar results were observed for the secondary outcome measures. At the end of the treatment phase, 80% (12/15) of participants demonstrated reliable improvement, and 60% (9/15) of participants demonstrated remission from social anxiety. Moreover, 7% (1/15) of participants dropped out during treatment, and 7% (1/15) of participants declined to undergo the follow-up phase after completing the treatment. No serious adverse events occurred. On average, participants completed 94% of the modules released to them. Participant feedback was positive and highlighted areas of strength in treatment, and it included further suggestions to improve suitability for Japanese settings.

**Conclusions:**

Translated and culturally adapted iCT-SAD demonstrated promising initial efficacy and acceptability for Japanese clients with social anxiety disorder. A randomized controlled trial is required to examine this more robustly.

## Introduction

### Background

Social anxiety disorder (SAD) is one of the most common mental disorders, with an early age of onset, a typically chronic course, and considerable impact on individuals’ quality of life [1]. These features lead to a large economic burden from a societal perspective [2]. Individual face-to-face cognitive therapy (CT) and cognitive behavioral therapy (CBT) has consistently been shown to be efficacious for SAD in a range of randomized controlled trials [3]. It is recommended as the gold standard psychological treatment in clinical practice guidelines in multiple countries (eg, Canada, Germany, and Australia and New Zealand) [4-6]. Guidelines from the United Kingdom [7] and Japan [8] specifically recommend individual CT based on the Clark and Wells model or individual CBT based on the Heimberg model, both of which have been uniquely developed to understand and treat SAD [9,10]. Although it has been shown that people with mental health problems tend to prefer psychological treatment over pharmacotherapy [11], access to evidence-based face-to-face psychological treatments, including CBT, remains limited worldwide [12]. This is particularly true in Japan, where SAD is prevalent [13] and the efficacy of individual face-to-face CT for antidepressant-resistant SAD has been demonstrated [14,15]. However, client’s access to CBT services is severely limited, mainly because of the insufficient number of therapists [16,17].

Efforts to improve access to psychological treatments by training new therapists have been made worldwide [18-22], but the impact of expanded training can be improved further by applying therapist-guided internet interventions. For both clients and therapists, the main benefits of web-based psychological treatments include flexibility, convenience, accessibility, and reduced therapist time per client, which can result in improved treatment accessibility and sustainability [23]. These benefits are also crucial during unpredictable natural disasters and public health emergencies, such as the COVID-19 pandemic, when there are insufficient resources for and limited access to professional mental health care.

In the United Kingdom, internet-delivered CT for SAD (iCT-SAD) [24,25] has been developed as web-based implementation of the face-to-face CT protocol that is based on the Clark and Wells cognitive model [9]. The face-to-face CT protocol has shown high efficacy in several randomized controlled trials [15,26-29]. iCT-SAD is a therapist-guided internet-delivered program, which follows a modular structure and replicates the key treatment components of face-to-face CT. The original English-language version of iCT-SAD showed strong efficacy in the treatment of SAD across studies in the United Kingdom and Hong Kong (within-group pre- to posttreatment Cohen *d* effect sizes for social anxiety >1.6) and was well accepted by clients (eg, dropout rates <10%) [24,25,30,31]. Randomized controlled trials indicated that iCT-SAD was superior to waitlist control conditions [24,30] and that clinical outcomes were comparable with that of face-to-face CT while requiring substantially less therapist time per client [24]. The results from these trials also demonstrated that the pre- and posttreatment effect sizes between the United Kingdom and Hong Kong were similar, suggesting the possibility of transporting iCT-SAD from one culture to another without substantial loss of efficacy. However, the trial conducted in Hong Kong used the English version of iCT-SAD without modification of the treatment content and treated only English-speaking residents in Hong Kong. Thus, it is still unknown whether iCT-SAD can retain its high efficacy once the treatment contents have been translated and adapted to a different culture.

### Objective

To make iCT-SAD more suitable for the Japanese context, we translated and adapted the treatment content and materials from English to Japanese [32]. We initially translated and back-translated the treatment materials within iCT-SAD, incorporating minor cultural adaptations where appropriate. The Japanese treatment material was then evaluated using a guided self-study approach with 6 Japanese SAD clients, where electronic versions of the Japanese modules were emailed and used independently by clients between 14 weekly in-person treatment sessions with their therapist. On the basis of the feedback from participants and the positive clinical outcomes observed in this guided self-study treatment (pre- to posttreatment Hedges *g*=2.31), it was concluded that the iCT-SAD treatment content was successfully translated and culturally adapted.

In this study, we embedded the finalized treatment material into a fully web-based Japanese iCT-SAD program. The main objective was to examine the preliminary efficacy and acceptability of the translated and culturally adapted iCT-SAD in Japanese clinical settings through a multicenter, single-arm, pre- to posttreatment study design. We also aimed to understand whether the UK and Hong Kong findings [24,25,30,31] could be replicated with the Japanese iCT-SAD program delivered to Japanese clients.

## Methods

### Study Design

This study used a single-group pre- to posttreatment design. Participant recruitment began in July 2021, and follow-up for all participants was completed by November 2022.

### Participants

A total of 15 participants with SAD were recruited from 5 medical institutions across Japan: the Fukunaga Internal and Neuropsychiatry Clinic (Miyazaki), Wakakusa Hospital (Miyazaki), Higashi-Omiya Mental Health Clinic (Saitama), Chiba University Hospital (Chiba), and Gokiso Mental Health Clinic (Nagoya).

All participants were receiving usual psychiatric care at the time of recruitment and had been doing so for an average of 11.6 (SD 12.1) months without substantial improvement in SAD symptoms. Each participant, along with their psychiatrist, had agreed that additional treatment was required. For participants’ safety and risk monitoring, they were asked to continue their regular appointments with their psychiatrist at their local medical institution in addition to working on iCT-SAD with the study therapist. We set this procedure because Japanese government regulations and implementation guidelines require that telemedicine be combined with regular face-to-face ambulatory care.

The inclusion criteria for this study were as follows: the participant must have a primary diagnosis of SAD according to the Diagnostic and Statistical Manual of Mental Disorders, Fifth Edition (DSM-5); be aged ≥18 years; be fluent in Japanese; have access to the internet via an appropriate device (eg, smartphone, tablet, and desktop or laptop computer); and be able to regularly visit their primary psychiatrist during the study. The following exclusion criteria were used: the presence of psychosis, bipolar disorder, or antisocial personality disorder; active suicidal ideation with intent or plan; intellectual disability or intellectual developmental disorder; current alcohol or substance dependence; and currently receiving any other structured psychological intervention or previously received CBT for SAD.

The diagnostic assessment based on the DSM-5 criteria was performed by an experienced psychiatrist at the study institution. During the study eligibility assessment, the study therapist further confirmed the DSM-5 diagnostic classification made by the psychiatrist and conducted a risk assessment interview.

### Intervention

The participants received iCT-SAD in combination with usual care. iCT-SAD is a therapist-guided internet intervention developed in the United Kingdom [24,25] based on the Clark and Wells cognitive model of SAD [9]. It implements all procedures from the face-to-face CT-SAD protocol [26] in a web-based format. iCT-SAD follows a modular structure, including 8 core modules that clients are encouraged to complete in the first 2 weeks to promote engagement and motivation. Thereafter, treatment is tailored to the individual concerns of each client using a range of specific modules covering common fearful beliefs or problems.

**Figure 1:**
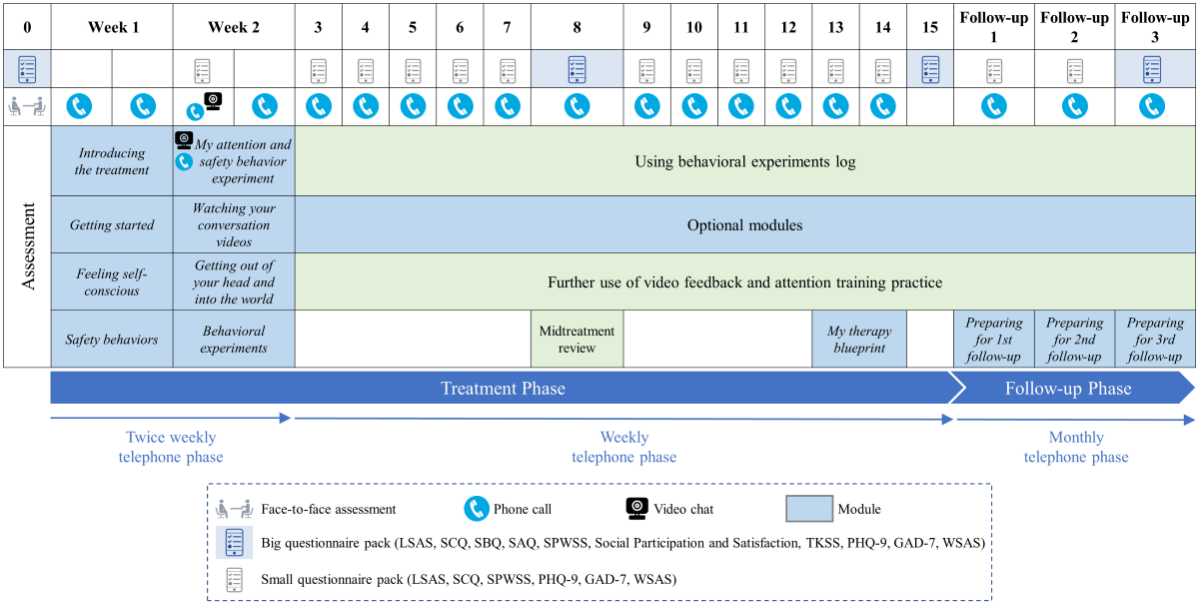
Overview of the internet-delivered cognitive therapy for social anxiety disorder treatment procedure. GAD-7: Generalized Anxiety Disorder Questionnaire 7-item; LSAS: Liebowitz Social Anxiety Scale; PHQ-9: Patient Health Questionnaire 9-item; SAQ: Social Attitudes Questionnaire; SBQ: Social Behavior Questionnaire; SCQ: Social Cognitions Questionnaire; SPWSS: Social Phobia Weekly Summary Scale; TKSS: Taijin Kyofusho Scale; WSAS: Work and Social Adjustment Scale.

iCT-SAD includes secure videoconferencing with recording functionality to facilitate the attention and safety behavior experiment and video feedback (more information on these techniques can be obtained in the study by Clark et al [26]), as well as *virtual audiences*, where clients can practice giving presentations. Clients were also encouraged to do several behavioral experiments each week. Therapists support their clients via telephone calls, asynchronous and SMS text messaging, and occasional video calls via webcam. In total, 2 telephone calls were scheduled per week in the first 2 weeks of treatment to support initial engagement, followed by a weekly phone call until the end of the 14-week treatment phase. In the follow-up phase, up to 3 booster phone calls were scheduled at monthly intervals. Each call lasted approximately 20 minutes and was used to review the client’s questionnaires, review progress with treatment modules and behavioral experiments, and plan for the coming week. Figure 1 provides an overview of the treatment structure (further details can be obtained in the study by Clark et al [24]).

To transport iCT-SAD to the Japanese context, the treatment content was translated from English into Japanese, and several minor adaptations were made to better suit the Japanese cultural context [32]. Adaptations were grouped into 4 categories (*Linguistics and Metaphors*, *Social Systems*, *Social Behaviors*, and *Familiarity*). *Linguistics and Metaphors* encompassed adaptations made because of differences in the way words, idioms, and metaphors are used. *Social Systems* referred to adaptations made because of differences in legal, educational, and health care systems. *Social Behavior* comprised adaptations made because of differences in the way people think and behave in social situations and what people expect of others in social situations. *Familiarity* included adaptations made so that names, songs, and products were more familiar to the Japanese people. One example from *Social Behavior* is that a suggested behavioral experiment in the original English version involved having a conversation about blushing within earshot of someone else on a bus or train to see how others respond. However, it is culturally inappropriate in Japan to talk loudly (or talk on the phone) on public transport, so adapting the experiment to take place while waiting for a bus or train is more suitable; the study of Yoshinaga et al [32] provides more details and further examples.

Following translation and cultural adaptation, we embedded the new content into a fully Japanese iCT-SAD web-based program, including audio and video materials.

### Therapists, Training, and Supervision

The study therapists consisted of 7 Japanese mental health professionals with experience in the use of individual face-to-face CT for SAD (clinical psychologists: n=3, 43%; registered nurses: n=3, 43%; and psychiatrist: n=1, 14%). Study therapists were mainly male (n=5, 71%), with a mean age of 39.7 (SD 4.6) years and an average of 11.5 (SD 2.3) years of postqualification clinical experience and 10.0 (SD 5.5) years of experience in providing CBT at the beginning of the study.

All study therapists received training in iCT-SAD, which was led by the second author (GRT), who has extensive experience in iCT-SAD. The study therapists initially attended a 2-day workshop in Japan, covering site navigation and functionality, structure and timing, module content, therapist communication, the implementation of key techniques, and troubleshooting. Each therapist was provided with test accounts to allow them to view the site from both client and therapist perspectives and to practice using the site during and after the workshop. To reinforce learning from the workshop, study therapists also referred to free therapist resources on CT for SAD and iCT-SAD [33]. These included workshop recordings, video illustrations of key techniques, and other therapist support materials (including adaptations for remote delivery). As the start of participant recruitment was delayed by the COVID-19 pandemic and its related restrictions, there was a time gap between the workshop and the implementation of iCT-SAD; thus, we implemented an additional 90-minute web-based refresher training before the enrollment of the first participant.

The first author (NY) provided weekly group supervision to the study therapists, which focused on reviewing clinical progress and planning sessions. NY also received individual supervision for his cases and *supervision of supervision* from GRT weekly to ensure that the implementation was consistent with the UK and Hong Kong studies.

### Outcomes

#### Preliminary Efficacy

The principal assessment points were baseline (week 0), midtreatment (week 8), posttreatment (week 15; primary assessment point), and follow-up (week 26). At these assessment points, participants were asked to complete the set of outcome measures listed in the following paragraphs. A subset of these questionnaires was also administered weekly during the main treatment phase to track clinical progress and tailor the intervention to the client. All measures were completed using the iCT-SAD program.

The primary outcome measure was the self-report version of the Liebowitz Social Anxiety Scale (LSAS) [34,35], which is one of the most commonly used scales for assessing the severity of social anxiety. The LSAS is a 24-item measure that consists of 2 subscales: fear and avoidance of performance and social interactional situations. Each item is rated on a 4-point Likert scale, ranging from 0 (*no fear* or *never avoid*) to 3 (*severe fear* or *usually avoid*).

The secondary outcomes included the following measures of social anxiety processes, *taijin kyofusho* (the fear of offending others), and general mood and functioning.

- Social anxiety process measures: central processes in the cognitive model of SAD were assessed using the Social Cognitions Questionnaire (SCQ) [36], which has frequency and belief subscales; Social Behavior Questionnaire (SBQ) [36]; Social Attitudes Questionnaire (SAQ) [36]; and Social Phobia Weekly Summary Scale (SPWSS) [36]. We also assessed participation in social activities and satisfaction with relationships using the Social Participation and Satisfaction scale [37].
- *Taijin kyofusho* measure: *taijin kyofusho* has frequently been discussed as a culture-specific expression of SAD unique to East Asia, characterized by the fear of offending others by one’s appearance or behavior [38]. It was assessed using the Taijin Kyofusho Scale (TKSS) [39], which consists of 31 items related to common concerns (eg, “I am afraid that when talking with others my trembling voice will offend them” and “I am afraid that eye to eye contact with other people will offend them”). Culturally sensitive measures such as this may be particularly helpful in evaluating psychological interventions in different cultural contexts.
- General mood and functioning measures: we used the Patient Health Questionnaire 9-item (PHQ-9) [40,41] to assess the severity of depressive symptoms, the Generalized Anxiety Disorder Questionnaire 7-item scale (GAD-7) [42,43] for generalized anxiety symptoms, and the Work and Social Adjustment Scale (WSAS) [44,45] for functional impairment associated with a health problem.

We also monitored adherence to treatment, medication use, and the incidence of adverse events. Adverse events, defined as any unfavorable psychological, emotional, or behavioral occurrence in the study participants (regardless of whether they were causally related to iCT-SAD), were monitored by the study therapists and primary psychiatrists throughout the study. Of these, we defined severe adverse events as those leading to death, life-threatening events requiring some form of high-intensity treatment (eg, hospital admission), or enduring severe impairment or dysfunction. At midtreatment (week 8), posttreatment (week 15), and follow-up assessment points (week 26), the study therapists completed a web-based form on the occurrence of any adverse events based on a verbal report from each participant and their primary psychiatrist.

To define response to treatment, remission from SAD, and reliable deterioration, we used the criteria described in the previous studies of iCT-SAD [24,25,30,31], which were based on the Jacobson and Truax methodology [46]. Response was defined as a pre- to posttreatment improvement on the LSAS >31%, which is comparable with a Clinical Global Impression improvement subscore of 2, a psychiatric measure often used to define response [47]. Remission was defined as a drop in the LSAS score of at least 12 points, combined with a posttreatment score of ≤38. Reliable deterioration was defined as an increase on the LSAS of at least 12 points and was examined from baseline to posttreatment and from posttreatment to follow-up assessment points.

#### Acceptability

The acceptability of iCT-SAD was measured based on the rate of participant dropout from the treatment, the level of engagement with the program, and participants’ feedback about their experience with iCT-SAD.

Dropout was defined as the premature cessation of treatment before completing the planned number of therapy weeks according to the protocol and not owing to participants’ recovery. Previous studies of iCT-SAD observed low dropout rates of <10% [24,25,30,31]. As this was a study in a new cultural setting, we set the threshold that a dropout rate >20% may suggest problems with treatment acceptability.

The level of engagement with the program was defined as the rate of module completion (ie, the percentage of released modules that were completed by participants). A previous UK study using iCT-SAD [24] observed a module completion rate of 81%, indicating strong engagement. In this study, we defined a completion rate of ≥70% as reflecting a good level of engagement.

At the end of the follow-up phase, participants were invited to complete a web-based feedback survey about their experiences with the treatment. This included questions about their experience with different modules, treatment components, and therapist behavior, which were rated on a Likert scale from 0 (*not helpful at all*) to 5 (*extremely helpful*). It also asked about the amount of therapist contact, which was rated on a Likert scale from 0 (*too little contact*) to 5 (*too much contact*), with a score of 3 indicating *Just the right amount*. The participants were also asked to provide overall comments and suggestions for improvement. As an exploratory study in a new cultural setting, we planned to review individual ratings and comments rather than set specific mean thresholds to indicate acceptability.

### Analysis

Participant demographics, clinical characteristics, activity on the website, and categorical outcomes were analyzed descriptively. Changes in continuous outcomes were analyzed using linear mixed effects models, which have the advantage of accounting for nested data structures and data missing at random. The models included time point (baseline, midtreatment, posttreatment, and follow-up) as a categorical fixed factor and participant as a random factor to account for between-subject variation. All models used restricted maximum likelihood estimation and an unstructured covariance matrix. *Q*-*Q* plots indicated that the normality of residuals assumption was met for all models. Effect sizes (Cohen *d*) were estimated by dividing the adjusted difference by the baseline SD. Participant feedback was analyzed descriptively.

### Ethics Approval

All aspects of this study complied with the Declaration of Helsinki, and the study protocol was reviewed and approved by the Ethics Committee of the University of Miyazaki (reference O-0879). Written informed consent was obtained from all the participants. Participant data confidentiality was guaranteed, and all study data were deidentified.

## Results

### Participant Flow and Baseline Clinical Characteristics

A total of 16 potential participants were referred to the study, of which 1 (6%) participant was not eligible (major depressive disorder was a primary diagnosis) and was referred elsewhere. Of the remaining 15 participants who took part in the study and entered the treatment phase, 14 (93%) participants completed the treatment phase (n=1, 7%, participant dropped out in week 6; for the analysis, week 6 questionnaire scores for this participant were used as the midtreatment assessment point where available), and 13 (93%) completed the subsequent follow-up phase (n=1, 7%, participant declined to undertake the follow-up phase but completed the follow-up assessment).

Baseline demographic and clinical characteristics of the participants are presented in Table 1. All participants met a primary diagnosis of SAD according to the DSM-5, and almost half (7/15, 47%) of the participants had a comorbid diagnosis, primarily major depressive disorders (5/15, 33%). More than half of the participants (9/15, 60%) were taking concurrent antidepressant medication at baseline. Notably, 87% (13/15) of participants had previously been treated with at least 1 course of selective serotonin reuptake inhibitor or serotonin-noradrenaline reuptake inhibitor treatment without substantial improvement in social anxiety; in other words, they exhibited resistance to gold standard pharmacological treatment for SAD at the beginning of the study [48].

**Table 1:** Baseline demographic and clinical characteristics (n=15).

Variable		Value
Sex (female), n (%)		10 (67)
Age (years), mean (SD)		27.3 (6.6)
**Marital status** **, n (%)**		
	Single	12 (80)
	Married	3 (20)
**Highest educational background** **, n (%)**		
	Junior high school	1 (7)
	Senior high school	6 (40)
	≥3 years of college or university	8 (53)
**Employment status** **, n (%)**		
	Full-time work	4 (27)
	Part-time work	1 (7)
	Student	5 (33)
	Sick leave	3 (20)
	Unemployed	2 (13)
Age of onset of SAD^a^ (years), mean (SD)		13.0 (4.1)
Duration of SAD (years), mean (SD)		14.3 (9.3)
**Comorbidity (current), n (%)**		
	Major depressive disorder	5 (33)
	Panic disorder	1 (7)
	Autism spectrum disorder	1 (7)
	None	8 (53)
Concurrent antidepressant medications (yes), n (%)		9 (60)
**Number of previous courses of SSRI^b^ or SNRI^c^ treatments, n (%)**		
	0	2 (13)
	1	7 (47)
	≥2	6 (40)

^a^SAD: social anxiety disorder.

^b^SSRI: selective serotonin reuptake inhibitor.

^c^SNRI: serotonin-noradrenaline reuptake inhibitor.

### Participant Use of iCT-SAD and Therapist Activity

Across the treatment phase, participants spent an average of 15.2 (SD 0.9) weeks, during which they logged into the program for a mean of 43.7 (SD 13.3) hours.

Therapists conducted a mean of 15.4 (SD 1.1) phone calls per participant, with a mean duration of 27.7 (SD 5.7) minutes for each call. They also made a mean of 1.3 (SD 0.5) video calls per participant, with a mean duration of 90.1 (SD 22.5) minutes for each call. Additionally, they sent an average of 48.8 (SD 17.4) asynchronous or SMS text messages to each participant. The total amount of direct live communication with each participant was therefore an average of 8.9 hours (equal to 538.6 minutes, SD 1.9 hours) during the treatment phase, with an additional 1.2 hours (70.1 minutes, SD 0.3 hours) during the follow-up phase. This is substantially less than the 18 to 21 hours required to deliver a course of face-to-face CT for SAD [15,26,27,29].

### Outcomes

#### Preliminary Efficacy

The results for the continuous outcomes are presented in Table 2. Significant improvements were observed on the primary outcome measure (LSAS; *P*<.001), indicating a reduction in social anxiety symptoms. All secondary outcomes showed significant changes over the time period examined, indicating reductions in symptoms of depression, generalized anxiety, and *taijin kyofusho*; reductions in processes linked to the maintenance of social anxiety (negative social cognitions, safety behaviors, and unhelpful social assumptions); increases in social participation and satisfaction; and a reduction in the functional impact of social anxiety. Effect size estimates (Cohen *d*) for the LSAS were 3.66 at posttreatment and 4.05 at follow-up, which are considered large. The pre- to posttreatment effect size estimates for the secondary outcomes ranged between 0.68 and 3.31.

Of the 15 participants, 12 (80%) demonstrated response to treatment, and 9 (60%) demonstrated remission from social anxiety. None of the participants showed reliable deterioration between the pre- and posttreatment assessments. Moreover, 7% (1/15) of participants showed reliable deterioration between posttreatment and follow-up assessments. This participant had declined to undergo the follow-up phase of the treatment.

Of the 9 participants taking antidepressant medications at baseline, 2 (22%) showed a decrease in dosage at posttreatment; 6 (67%) remained at the same dosage; and 1 (11%) showed an increase. In addition, 2 participants started taking antidepressants during the study. A sensitivity analysis excluding the 3 participants who increased dosages or started antidepressants during the study showed a similar pre- to posttreatment effect size (Cohen *d*=3.35) to the main analysis.

No severe adverse events were observed during the study. Adverse events reported were either unrelated to the treatment (eg, feeling exhausted owing to increased demands at work and feeling unwell owing to an adverse reaction to the COVID-19 vaccine) or reflected normal symptom fluctuation during treatment (eg, increased anxiety symptoms before a challenging behavioral experiment). The latter category included the participant who dropped out and the participant who declined to undertake the follow-up phase. Both participants later indicated that one of the reasons for this was finding the interaction with their therapist anxiety provoking, especially when planning and conducting challenging behavioral experiments.

**Table 2:** Unadjusted means, SDs, adjusted differences, and effect sizes for the intent-to-treat sample (n=15).

Measure and time point		Unadjusted mean (SD)	Adjusted difference (95% CI)	*P* value	Within-group effect size, Cohen *d* (95% CI)^a^
**LSAS^b^**					
	Pretreatment	84.6 (14.2)	N/A^c^	N/A	N/A
	Midtreatment	59.1 (17.0)	25.5 (14.4 to 36.7)	<.001	1.80 (1.01 to 2.59)
	Posttreatment	31.7 (21.6)	51.9 (40.4 to 63.3)	<.001	3.66 (2.85 to 4.46)
	Follow-up	26.1 (24.0)	57.5 (46.1 to 68.9)	<.001	4.05 (3.25 to 4.86)
**SCQ^d^ frequency**					
	Pretreatment	3.1 (0.7)	N/A	N/A	N/A
	Midtreatment	2.2 (0.8)	0.9 (0.5 to 1.2)	<.001	1.25 (0.74 to 1.76)
	Posttreatment	1.5 (0.4)	1.5 (1.2 to 1.9)	<.001	2.24 (1.72 to 2.77)
	Follow-up	1.5 (0.5)	1.6 (1.2 to 1.9)	<.001	2.31 (1.78 to 2.83)
**SCQ** **belief**					
	Pretreatment	55.5 (18.2)	N/A	N/A	N/A
	Midtreatment	29.8 (17.6)	25.7 (16.6 to 34.8)	<.001	1.41 (0.91 to 1.91)
	Posttreatment	13.2 (12.0)	41.6 (32.3 to 50.9)	<.001	2.29 (1.77 to 2.80)
	Follow-up	9.9 (12.2)	45.0 (35.7 to 54.3)	<.001	2.47 (1.96 to 2.98)
**SBQ^e^**					
	Pretreatment	43.3 (8.5)	N/A	N/A	N/A
	Midtreatment	33.3 (9.0)	9.9 (3.6 to 16.2)	.003	1.16 (0.43 to 1.90)
	Posttreatment	24.4 (10.6)	18.8 (12.6 to 25.1)	<.001	2.21 (1.48 to 2.95)
	Follow-up	20.0 (10.7)	22.9 (16.5 to 29.3)	<.001	2.69 (1.94 to 3.44)
**SAQ^f^**					
	Pretreatment	208.7 (24.2)	N/A	N/A	N/A
	Midtreatment	160.2 (35.2)	48.6 (29.2 to 67.9)	<.001	2.01 (1.21 to 2.80)
	Posttreatment	128.6 (46.9)	80.2 (60.9 to 99.5)	<.001	3.31 (2.52 to 4.11)
	Follow-up	122.4 (44.5)	86.4 (67.1 to 105.7)	<.001	3.57 (2.77 to 4.37)
**SPWSS^g^**					
	Pretreatment	25.8 (7.9)	N/A	N/A	N/A
	Midtreatment	21.2 (7.3)	4.6 (0.1 to 9.1)	.04	0.58 (0.02 to 1.15)
	Posttreatment	12.4 (7.2)	12.7 (8.1 to 17.2)	<.001	1.61 (1.03 to 2.19)
	Follow-up	12.3 (9.7)	12.7 (8.2 to 17.3)	<.001	1.62 (1.04 to 2.20)
**Social participation**					
	Pretreatment	34.2 (12.0)	N/A	N/A	N/A
	Midtreatment	51.8 (16.0)	−17.7 (−23.9 to −11.5)	<.001	1.48 (0.96 to 2.00)
	Posttreatment	56.4 (11.8)	−22.3 (−28.5 to −16.1)	<.001	1.87 (1.35 to 2.39)
	Follow-up	52.9 (18.7)	−18.8 (−25.0 to −12.5)	<.001	1.57 (1.05 to 2.09)
**Social satisfaction**					
	Pretreatment	14.9 (8.0)	N/A	N/A	N/A
	Midtreatment	20.5 (9.2)	−5.5 (−8.1 to −2.9)	<.001	0.69 (0.36 to 1.02)
	Posttreatment	22.9 (7.8)	−7.9 (−10.6 to −5.3)	<.001	0.99 (0.66 to 1.32)
	Follow-up	24.1 (8.6)	−9.2 (−11.8 to −6.5)	<.001	1.14 (0.82 to 1.47)
**TKSS^h^**					
	Pretreatment	136.8 (33.6)	N/A	N/A	N/A
	Midtreatment	109.2 (35.5)	27.0 (9.9 to 44.0)	.003	0.80 (0.30 to 1.31)
	Posttreatment	75.1 (25.0)	61.0 (44.0 to 78.1)	<.001	1.82 (1.31 to 2.33)
	Follow-up	75.6 (32.9)	60.6 (43.6 to 77.7)	<.001	1.80 (1.30 to 2.31)
**PHQ-9^i^**					
	Pretreatment	12.3 (5.6)	N/A	N/A	N/A
	Midtreatment	9.2 (5.4)	3.1 (0.9 to 5.2)	.007	0.55 (0.16 to 0.94)
	Posttreatment	6.0 (5.6)	6.0 (3.7 to 8.0)	<.001	1.07 (0.67 to 1.47)
	Follow-up	5.9 (6.2)	6.1 (3.9 to 8.3)	<.001	1.09 (0.69 to 1.49)
**GAD-7^j^**					
	Pretreatment	9.3 (5.3)	N/A	N/A	N/A
	Midtreatment	6.7 (3.5)	2.6 (0.6 to 4.6)	.01	0.49 (0.11 to 0.86)
	Posttreatment	3.1 (2.5)	6.9 (4.0 to 8.1)	<.001	1.14 (0.76 to 1.52)
	Follow-up	3.0 (2.7)	6.2 (4.2 to 8.3)	<.001	1.17 (0.79 to 1.55)
**WSAS^k^**					
	Pretreatment	13.5 (8.5)	N/A	N/A	N/A
	Midtreatment	11.8 (9.7)	1.7 (−2.1 to 5.4)	.37	0.20 (−0.24 to 0.64)
	Posttreatment	7.3 (8.3)	5.8 (1.9 to 9.6)	.004	0.68 (0.23 to 1.13)
	Follow-up	6.8 (9.3)	6.3 (2.4 to 10.1)	.002	0.74 (0.28 to 1.19)

^a^Within-group effect sizes represent change from baseline using the adjusted difference scores at each time point.

^b^LSAS: Liebowitz Social Anxiety Scale.

^c^N/A: not applicable.

^d^SCQ: Social Cognitions Questionnaire (mean scores).

^e^SBQ: Social Behavior Questionnaire.

^f^SAQ: Social Attitudes Questionnaire.

^g^SPWSS: Social Phobia Weekly Summary Scale.

^h^TKSS: Taijin Kyofusho Scale.

^i^PHQ-9: Patient Health Questionnaire 9-item.

^j^GAD-7: Generalized Anxiety Disorder Questionnaire 7-item.

^k^WSAS: Work and Social Adjustment Scale.

#### Acceptability

As described earlier, 7% (1/15) of participants dropped out during the treatment phase. This reflected a good overall level of treatment acceptability. The core modules were released to, and completed by, all the participants. On average, 13.0 (SD 3.6) additional modules were released and 11.8 (SD 3.8) optional modules were completed. On average, participants completed 94% of the modules released to them, indicating a strong level of engagement with the program. They also completed an average of 17.6 (SD 7.8) behavioral experiments.

A total of 87% (13/15) of participants, all of whom completed the treatment and follow-up phases, responded to the feedback survey. The participant who dropped out and the participant who declined the follow-up phase did not respond to invitations to submit feedback. As presented in Table 3, respondents’ mean ratings on a Likert scale from 0 (*not helpful at all*) to 5 (*extremely helpful*) indicated that the core modules of *Safety behaviors*, *Behavioral experiments*, and *Feeling self-conscious* were perceived as the most helpful. The treatment components rated as the most helpful were phone calls, written example vignettes, and behavioral experiment logs. The virtual audience feature received the lowest mean rating, although it had greater variability, and all other treatment components received ratings >3.62. The most helpful therapist behaviors were making suggestions for behavioral experiments, explaining things in the program, and providing reminders (eg, about working on the site, experiments, or completing questionnaires). Respondents’ comments also highlighted the helpfulness of the therapist having an *affirming attitude*, focusing on the client’s strengths, flexibility in approach, and openness to questions. Respondents also rated the amount of contact from their therapist on a Likert scale, ranging from 0 (*too little contact*) to 5 (*too much contact*), with a score of 3 indicating *Just the right amount*. The mean rating was 3.23 (SD 0.44).

Respondents’ suggestions for improvement included fixing technical issues such as delays in receiving 2-factor authentication codes to access the site and including more video illustrations or testimony involving Japanese clients. Some respondents also noted difficulty in finding time to work on the treatment site regularly because of their busy schedules.

**Table 3:** Mean ratings on a Likert scale for participant experience with the therapy (n=13)^a^.

Item		Values, mean (SD)
**Core modules^b^**		
	Introducing the treatment	4.46 (0.66)
	Getting started	4.38 (0.77)
	Feeling self-conscious	4.69 (0.48)
	Safety behaviors	4.85 (0.38)
	My attention and safety behavior experiment	4.54 (0.66)
	Watching your conversation videos	4.46 (0.78)
	Getting Out of your head and into the world	4.38 (0.77)
	Behavioral experiments	4.77 (0.60)
**Treatment components^b^**		
	Video examples	4.00 (0.71)
	Written vignettes examples	4.46 (0.66)
	Testimonies from previous clients	3.85 (0.69)
	Street surveys	3.85 (1.07)
	Attention training exercises (video and audio)	4.00 (0.82)
	Behavioral experiment log	4.31 (0.63)
	Webcam	3.69 (0.95)
	Library	3.77 (1.24)
	My model	3.62 (0.65)
	Virtual audiences	2.62 (1.66)
	Messaging function (e-mails) within website	3.92 (1.12)
	SMS text messages	3.92 (1.04)
	Phone calls	4.69 (0.63)
	Webcam chats	4.23 (0.83)
**Therapist behaviors^b^**		
	Suggestions for new behavioral experiments	4.62 (0.65)
	General encouragement	4.54 (0.78)
	Clarification for completed modules	4.31 (0.85)
	Clarification for completed experiments	4.38 (0.77)
	Helping me reexamine my beliefs	4.23 (1.01)
	Explaining things in the internet program	4.54 (0.78)
	Reminders (eg, to log on, to complete questionnaires, and to complete behavioral experiments)	4.54 (0.66)
The amount of therapist contact^c^		3.23 (0.71)

^a^A total of 2 of the 15 participants who dropped out of treatment or declined to undergo the follow-up phase did not respond to invitations to submit feedback.

^b^Rated on a Likert scale from 0 (not helpful at all) to 5 (extremely helpful).

^c^Rated on a Likert scale from 0 (too little contact) to 5 (too much contact), with a score of 3 indicating Just the right amount.

### Benchmarking Against UK and Hong Kong Efficacy

Table 4 presents a comparison of the results of this study with those of previous UK and Hong Kong studies [24,25,30,31]. Participants in Japan showed at least as much improvement as those in the United Kingdom and Hong Kong. The larger effect size relative to other studies may have resulted from the higher baseline severity and lower baseline SD in this sample.

**Table 4:** Comparison of results with UK and Hong Kong studies using internet-delivered cognitive therapy for social anxiety disorder (iCT-SAD).

	Stott et al [25], 2013	Clark et al [24], 2022	Thew et al [31], 2019	Thew et al [30], 2022	This study
Study type	Single-arm trial	RCT^a^ (iCT-SAD arm)	Single-arm trial	RCT (iCT-SAD arm)	Single-arm trial
Location	United Kingdom	United Kingdom	Hong Kong	Hong Kong	Japan
Participants, N	11	49	6	22	15
LSAS^b^ at baseline, mean (SD)	80.0 (24.6)	77.7 (17.7)	75.8 (30.4)	76.1 (19.7)	84.6 (14.2)
LSAS at posttreatment, mean (SD)	39.8 (30.1)	32.2 (19.6)	22.0 (21.0)	21.1 (21.0)	31.7 (21.6)
Effect size (pre- to posttreatment Cohen *d*)^c^	1.63	2.57	1.77	2.79	3.73
Response rate (%)^d^	81.8	85.4	83.3	95.5	80
Remission rate (%)^d^	63.6	75	83.3	86.4	60

^a^RCT: randomized controlled trial.

^b^LSAS: Liebowitz Social Anxiety Scale.

^c^For comparability, all effect sizes shown are within-group and are calculated using raw unadjusted mean LSAS scores and the baseline SD, as these values are available in all papers.

^d^The criteria to define response and remission were the same across studies and relate to the posttreatment assessment.

## Discussion

### Principal Findings and Comparison With Prior Work

This study aimed to examine the preliminary efficacy and acceptability of culturally adapted Japanese iCT-SAD in Japanese clinical settings and to understand whether the UK and Hong Kong findings [24,25,30,31] could be replicated when translated and culturally adapted iCT-SAD was used in the Japanese context. The results yielded 2 key findings. First, the treatment led to significant improvements on all outcome measures, and these improvements were maintained during the follow-up phase. Although controlled studies are needed for more robust comparisons, the within-group LSAS effect size in this pilot trial was comparable with those obtained in studies of the English-language program in the United Kingdom and Hong Kong [24,25,30,31]. Furthermore, in line with the previous UK and Hong Kong studies, Japanese iCT-SAD required less therapist contact time per client (8.97 hours) compared with face-to-face CT (18-21 hours), representing a time saving of 50% to 58% compared with an equivalent treatment. This provides an initial indication that previous findings can be successfully replicated when translated and culturally adapted iCT-SAD is used in a different cultural setting. Second, the acceptability of the intervention was demonstrated by most participants completing the treatment, showing a strong level of engagement in the program, and providing positive feedback.

The baseline clinical characteristics of our participants were slightly different from those observed in the previous UK and Hong Kong studies. This sample had a relatively higher severity of social anxiety and depressive symptoms at baseline. However, this severity was similar to that observed in previous studies of SAD in Japan [15,49]. In addition, many participants in this study were taking antidepressants at baseline and had previously been treated with at least 1 selective serotonin reuptake inhibitor or serotonin-noradrenaline reuptake inhibitor. This is commonly seen among Japanese clinical samples seeking psychological treatment because pharmacotherapy has historically been more common in Japan [21,49-52]. Therefore, this sample appears to be representative of clinical SAD samples in Japan.

The findings of this study are consistent with those of other studies of culturally adapted CBT internet interventions, which have typically found that these interventions have been well accepted by participants with positive outcomes for problems including SAD [53-56], depression [57-59], and insomnia [60,61]. However, it has been highlighted that the exact details of the cultural adaptations made are not always provided and could be made more explicit in future studies [32].

A total of 33% (5/15) of the participants had a comorbid major depressive disorder at baseline; 4 (80%) of these 5 participants successfully worked on and completed iCT-SAD, showing improvements in both SAD and depression. This is consistent with previous studies on CT-SAD and iCT-SAD, which have indicated that when depression symptoms are secondary to SAD, the provision of SAD-focused treatment tends to also relieve depression [15,24,26-30]. The iCT-SAD program contains an optional *Managing my mood* module, which can be provided to clients who require further support around this or in situations where low mood may be reducing motivation to work on other aspects of treatment. The program also administers the PHQ-9 weekly, which allows the therapist to monitor symptoms of depressed mood and clinical risk, and if warranted based on their clinical judgment, to take immediate steps to support clients’ safety. Clinicians who are assessing comorbid SAD and depression are encouraged to inquire about the chronology of these 2 problems, current risk, and the client’s goals for therapy when making treatment-related decisions.

As client adherence is a key determinant of the effectiveness of internet treatment for anxiety and depressive disorders [62], we must caution that 1 participant dropped out of treatment and 1 participant declined to undertake the follow-up phase because they found the interaction with their therapist anxiety provoking. It is notable that this is not something that they were able to express directly to their therapist and reflects a broader challenge in treating SAD as clients may view the therapist as a *phobic object* [63] and therefore use safety behaviors such as avoiding sharing their opinions. It is possible this may be particularly common in Japanese or other East Asian contexts because Japanese clients tend to view the therapist as an authority figure and deify the therapist [64]. Early modules in iCT-SAD aim to elicit potential concerns from clients about treatment in general and treatment in a web-based format, which can facilitate discussions with the therapist and address potential barriers to engagement. However, the results of this study highlight the need for therapists to keep this in mind throughout treatment and to take opportunities to ask directly about treatment-related concerns.

### Limitations

The main limitations of this study include (1) the small number of participants, (2) no control group for comparison, (3) three participants having started or increased antidepressants during the study, and (4) participant feedback not being based on the whole study sample. Furthermore, (5) iCT-SAD was provided in combination with usual care, which means that the specific effects of iCT-SAD alone cannot be fully evaluated using this study design. The participants were already receiving usual care on a regular basis from their psychiatrist at the time of recruitment. Although this means that the improvements observed cannot be due to the effects of initiating work with a psychiatrist, it could perhaps be that other aspects of continued psychiatrist contact, or the combination with iCT-SAD, contributed to the improvements observed in this study. Notwithstanding these limitations, this study yielded promising findings that warrant further investigation in a larger randomized controlled trial.

### Conclusions

This pilot trial suggests that translated and culturally adapted iCT-SAD shows promising initial efficacy and acceptability in the treatment of SAD with Japanese clients. This is evidenced by the excellent clinical outcomes obtained in treatment using the Japanese iCT-SAD modules, the low dropout rate from the treatment, a strong level of participant engagement in the program, and the positive feedback provided by participants, all of which are comparable with what was observed in the English-language program in the UK and Hong Kong studies. iCT-SAD shows promise as a way of providing evidence-based psychological therapies for a wide range of people experiencing social anxiety in Japan. Future studies should build on these preliminary findings by conducting a randomized controlled trial with larger samples.

## Abbreviations

CBT: cognitive behavioral therapy
CT: cognitive therapy
DSM-5: Diagnostic and Statistical Manual of Mental Disorders, Fifth Edition
GAD-7: Generalized Anxiety Disorder Questionnaire 7-item
iCT-SAD: internet-delivered cognitive therapy for social anxiety disorder
LSAS: Liebowitz Social Anxiety Scale
PHQ-9: Patient Health Questionnaire 9-item
SAD: social anxiety disorder
SAQ: Social Attitudes Questionnaire
SBQ: Social Behavior Questionnaire
SCQ: Social Cognitions Questionnaire
SPWSS: Social Phobia Weekly Summary Scale
TKSS: Taijin Kyofusho Scale
WSAS: Work and Social Adjustment Scale

## Data Availability

Anonymized data from this paper are available for research purposes on request to the corresponding author. Broader public data sharing for this study has been restricted by the Ethics Committee at the University of Miyazaki because the consent obtained from the participants does not cover the unlimited public sharing of the data.
